# Enhanced Electrochemiluminescence by Nanocatalyst-Supported Nanochannel–Surfactant Micelle Assembly for Ultrasensitive Detection of Rifampicin

**DOI:** 10.3390/bios16050236

**Published:** 2026-04-23

**Authors:** Jiahui Lin, Zhongping Mao, Fei Yan

**Affiliations:** School of Chemistry and Chemical Engineering, Zhejiang Sci-Tech University, Hangzhou 310018, China; 2023221002031@mails.zstu.edu.cn (J.L.); 2025211001048@mails.zstu.edu.cn (Z.M.)

**Keywords:** electrochemiluminescence, luminol-dissolved oxygen, confined surfactant micelles, composite nanocatalyst, rifampicin

## Abstract

Developing an ultrasensitive electrochemiluminescence (ECL) detection platform remains challenging due to the limited enrichment efficiency of ECL emitters and co-reactants at the electrode interface, as well as the insufficient catalytic enhancement of co-reactant conversion. Moreover, simultaneous in situ analyte enrichment and efficient anti-interference capability are often difficult to achieve in a single sensing interface. Herein, a new ECL platform was developed based on nanocatalyst-supported nanochannel-confined surfactant micelle (SM) system, which integrates an enhanced luminol-dissolved oxygen (DO) ECL response for the ultrasensitive detection of antibiotic rifampicin (RIF). A nanocomposite comprising nitrogen-doped graphene quantum dots and a molybdenum disulfide nanosheet (NGQDs@MoS_2_) was modified on an indium tin oxide (ITO) electrode. This nanocomposite layer catalyzed the oxygen reduction reaction (ORR), boosting the co-reactant efficiency of DO. Vertically ordered mesoporous silica film filled with surfactant micelles (SM@VMSF) was subsequently grown in situ on the NGQDs@MoS_2_ surface. The hydrophobic micelles enable the simultaneous enrichment of luminol, DO, and RIF. Integrating the triple-enrichment effect of surfactant micelles with the high electrocatalytic effect of NGQDs@MoS_2_ nanocomposite results in significant ECL enhancement of the luminol–DO. SM@VMSF also provides an excellent molecular sieving effect, endowing the sensor with high anti-interference capability and stability. RIF quenches the ECL signal by consuming superoxide anion radicals, enabling sensitive detection. Detection of RIF was established with a high sensitivity (2927 a.u. per nM) wide linear range (10 pM to 10 μM) and a low limit of detection (LOD, 2.5 pM). The fabricated sensor exhibits good selectivity and high fabrication reproducibility (relative standard deviation, RSD, of 1.9%). Additionally, the determination of RIF in eye drops and seawater samples was realized. This work offers new insights for the design of high-performance ECL sensing interfaces and sensitive detection of RIF.

## 1. Introduction

Rifampicin (RIF), a widely used broad-spectrum antibiotic in clinical practice, exhibits potent antibacterial and anti-inflammatory activities [1]. Its action mechanism primarily involves blocking RNA synthesis and suppressing pathogenic microorganisms [2]. In addition to systemic administration, RIF is frequently incorporated into topical anti-infective formulations, including eye drops, to exert its therapeutic effects [3]. However, long-term, excessive, or inappropriate use of RIF may lead to adverse effects such as gastrointestinal disturbances, hepatotoxicity, and nephrotoxicity. Furthermore, the misuse of antibiotics poses potential risks of environmental contamination. Therefore, the accurate, sensitive, and efficient detection of RIF is of significant importance for both clinical monitoring and environmental assessment. Currently reported detection methods for RIF include high-performance liquid chromatography (HPLC) [4], electrochemical techniques [5], and fluorescence analysis [6]. However, these approaches usually require high instrument costs, long analysis times, and complex sample pretreatment. Rapid, sensitive, and user-friendly determination of RIF is highly desirable.

Electrochemiluminescence (ECL) analysis is powerful in pharmaceutical analysis [7], biosensing [8], food safety [9,10,11], clinical diagnostics [12,13,14], and environmental monitoring due to its advantages including low background signal, operational simplicity, wide dynamic range, and excellent spatiotemporal controllability [15]. The ECL intensity is highly related with the local concentration of ECL emitters and co-reactants at the electrode interface, as well as their reaction efficiency. To address this, various signal amplification strategies have been developed. On the one hand, nanocatalysts are introduced at the electrode interface to promote the co-reactant reaction and enhance reaction efficiency [16,17,18,19]. On the other hand, enrichment effects are employed to increase the local concentration of ECL emitters and co-reactants on the electrode surface, thereby achieving signal amplification [20,21,22]. The synergistic integration of catalytic enhancement and enrichment effects at the electrode interface provides an effective strategy for constructing a highly sensitive ECL sensing platform.

Two-dimensional (2D) layered materials are attractive because of their unique electronic structures and exceptional physicochemical properties [23,24,25,26]. Among them, molybdenum disulfide (MoS_2_) has shown great promise in electrocatalysis and sensing applications due to its layered structure, favorable electrocatalytic activity, moderate electrical conductivity, and advantages of natural abundance and low cost [27,28]. Bulk MoS_2_ is typically exfoliated into few-layer or monolayer nanosheets to increase surface area and active sites. However, pristine MoS_2_ nanosheets still suffer from limitations such as insufficient catalytic active sites and limited conductivity [29]. The use of functional zero-dimensional (0D) materials as intercalating agents, combined with ultrasonic-assisted exfoliation, enables the fabrication of well-dispersed and structurally stable nanocomposites with enhanced conductivity and catalytic performance, offering new opportunities for constructing high-performance electrochemical interfaces [30]. However, the direct modification of electrodes with nanomaterials often encounters low stability, including weak adhesion and susceptibility to detachment during prolonged use, which restricts their application in complex sample analysis [31]. Furthermore, ECL sensors are frequently required to analyze complex matrices (e.g., biological fluids, environmental samples, etc.), where macromolecules (e.g., proteins) and other electroactive species can undergo non-specific adsorption onto the electrode, resulting in surface fouling and compromised reliability. Therefore, enhancing interfacial stability, anti-fouling capability, and selectivity and improving catalytic activity is crucial for the development of high-performance ECL sensors.

Growing a vertically ordered mesoporous silica film (VMSF) on electrode surfaces represents a versatile interfacial modification strategy that integrates functional material immobilization and anti-fouling as well as anti-interference capacities [32,33,34,35]. Compared to disordered porous silica materials, VMSF possesses unique structural and functional properties [36,37,38]. VMSF is an ultrathin nanofilm with a tunable thickness ranging from 20 to 200 nm, featuring a nanochannel array that is oriented perpendicular to the underlying electrode, with uniform nanochannel diameters (typically 2–3 nm) and high density (up to 75,000 pores/μm^2^) [39,40,41]. Consequently, the open structure of VMSF ensures high diffusion performance. On the other hand, as the diameter of the nanochannels is similar to the Debye length, VMSF nanochannels exhibit molecular-level sieving capabilities based on size, electrostatic interactions, and hydrophilicity/hydrophobicity [42]. Additionally, during VMSF growth, surfactant micelles are employed as templates, and after film formation, hydrophobic surfactant assemblies (SMs) remain confined within the nanochannels, yielding a SM@VMSF composite architecture [43,44]. This unique structure not only facilitates the enrichment of hydrophobic ECL emitters or target molecules but also effectively reduces interference from hydrophilic species, providing an ideal platform for constructing highly selective sensors for hydrophobic analytes. Consequently, the combination of highly catalytic nanocomposites with SM@VMSF holds great promise for achieving the synergistic optimization of catalytic enhancement, selectivity improvement, and anti-fouling performance, thereby presenting a novel material design strategy for ECL sensing platforms.

In this work, a highly sensitive ECL sensor based on a SM@VMSF composite film and NGQDs@MoS_2_ nanocomposite was constructed on an ITO electrode for the detection of the antibiotic RIF. The NGQDs@MoS_2_ composite effectively enhances catalytic activity and promotes ORR to generate ROS, thereby amplifying the ECL response. Meanwhile, VMSF provides nanochannels for confining surfactant micelles (SMs). The SM@VMSF composite film integrates enrichment and anti-fouling functions, not only enriching the ECL emitter, co-reactant, and hydrophobic target RIF but also improving electrode anti-interference capability and stability in complex matrices. Through the synergistic integration of these components on the ITO electrode surface, a stable sensing interface was fabricated, enabling the sensitive detection of RIF. RIF consumes the key species (superoxide anion radicals) during the ECL emission process of the luminol–DO system, leading to a decrease in ECL. Based on this principle, RIF was detected. This work provides a rapid analysis of RIF and offers new insights into the application of VMSF film and 2D nanocomposites in the field of ECL sensing.

## 2. Materials and Methods

### 2.1. Chemicals and Materials

Tetraethoxysilane (TEOS, 99%), NaHCO_3_ (analytical grade-AR, 99.8%), NaOH (AR, 98%), tert-butyl alcohol (TBA, 99%), luminol (98%), p-benzoquinone (BQ, 99%), cetyltrimethylammonium bromide (CTAB, 99%), K_3_[Fe(CN)_6_] (99.5%), rifampicin (RIF, 99%), K_4_[Fe(CN)_6_] (99%), NH_3_·H_2_O (28%), hexaammineruthenium(III) chloride ([Ru(NH_3_)_6_]Cl_3_, 98%), glucose (Glu, AR), hydroxymethylferrocene (FcMeOH, 95%), uric acid (UA, AR, 99%), Na_2_HPO_4_·12H_2_O (AR, 99%), 1-aminopyrene (98%), ascorbic acid (AA), NaH_2_PO_4_·2H_2_O (AR, 99%), molybdenum disulfide (MoS_2_, 98%), NaCl (AR, 99.5%), NaNO_3_ (AR, 99%), KCl (AR, 99%), tetracycline (TC, 96%) and levofloxacin (LEV, 98%) were obtained from Aladdin Biochemical Technology Co., Ltd. (Shanghai, China).

### 2.2. Measurements and Instrumentations

X-ray photoelectron spectroscopy (XPS) was conducted on a K-Alpha photoelectron spectrometer (Thermo Scientific, Waltham, MA, USA). Transmission electron microscopy (TEM) and high-resolution TEM (HRTEM) images were captured using a HT7700 microscope (Hitachi, Tokyo, Japan) and a JEM-2100F field emission microscope (JEOL, Tokyo, Japan), respectively. Scanning electron microscopy (SEM) images were obtained with a Zeiss Sigma500 field emission microscope (Zeiss, Oberkochen, Germany). ECL measurements were performed on a MPI-E II ECL analyzer (Xi’an Remex Analytical Instrument Co., Xi’an, China). Cyclic voltammetry (CV) was carried out using an Autolab PGSTAT302N electrochemical workstation (Metrohm, Herisau, Switzerland). The scan rate was set at 100 mV·s^−1^. All electrochemical and ECL experiments employed a conventional three-electrode system: an Ag/AgCl electrode as the reference electrode, a platinum wire as the counter electrode, and bare or modified ITO electrodes as the working electrodes. Unless otherwise specified, the photomultiplier tube (PMT) voltage was 650 V during ECL measurements.

### 2.3. Synthesis of NGQDs

NGQDs were synthesized via a hydrothermal process using 1-aminopyrene as a precursor [45]. Specifically, the hydrothermal treatment of 1-aminopyrene solution (2 mg/mL in 0.4 M ammonia solution) was performed for 8 h at 200 °C. The obtained solution was dialyzed against ultrapure water for 24 h using a dialysis membrane (cut-off Mw 500 Da) to eliminate unreacted small molecules. The dialysate was filtered through a 0.22 μm membrane and lyophilized to obtain solid NGQDs.

### 2.4. Preparation of NGQDs@MoS_2_ Nanocomposite

The NGQDs@MoS_2_ nanocomposite was prepared via ultrasonication-assisted exfoliation employing NGQDs as an intercalation agent. Initially, MoS_2_ powder was dispersed in 50% ethanol and magnetically stirred (2 h) to yield a homogeneous suspension (7.5 mg/mL). Subsequently, NGQDs were introduced into the suspension, and the mixture was ultrasonically treated for 72 h (200 W power, 40 kHz frequency). Following ultrasonication, the mixture was centrifuged (7000 rpm, 20 min) and the supernatant was further centrifuged (13,000 rpm, 30 min). The precipitate was washed three times with ethanol. The final product was re-dispersed in 50 mL of 50% ethanol aqueous solution to obtain a NGQDs@MoS_2_ dispersion (0.5 mg/mL with respect to MoS_2_).

### 2.5. Fabrication of Different Electrodes

ITO conductive glass (2.5 × 5 cm) was immersed in 1 M NaOH solution for 12 h and cleaned using acetone, ethanol, and ultrapure water, respectively. After drying under nitrogen flow, the slides were cut into working electrodes (0.5 × 5 cm) for subsequent use. Modification of ITO electrodes with NGQDs@MoS_2_ was achieved by a drop-casting method. Specifically, 20 μL of NGQDs@MoS_2_ dispersion (0.05 mg/mL) was dropped on the ITO electrode and dried with an infrared lamp for 30 min, yielding the NGQDs@MoS_2_/ITO electrode. For comparison, the control electrodes (NGQDs/ITO and MoS_2_/ITO) were prepared by drop-casting the corresponding NGQDs or MoS_2_ solutions on the ITO electrodes, respectively.

Subsequently, VMSFs were rapidly grown on the NGQDs@MoS_2_/ITO via the electrochemically assisted self-assembly (EASA) method [46]. The as-prepared electrode, comprising CTAB SM and the nanochannel assay (SM in nanochannels, SM@VMSF/NGQDs@MoS_2_/ITO), was obtained. For control experiments, SM@VMSF on NGQDs or MoS_2_-modified ITO electrodes were also fabricated following the identical procedure.

### 2.6. ECL Detection of RIF

The as-prepared SM@VMSF/NGQDs@MoS_2_/ITO electrode was directly employed for RIF detection without any further activation or modification. PBS (0.01 M, pH 7.4) containing 100 μM luminol was the supporting electrolyte. CV (−1.0 to +0.8 V vs. Ag/AgCl) was applied to trigger ECL at a scan rate of 100 mV/s, with a scanning sequence of 0 V → −1.0 V → +0.8 V → 0 V, while simultaneously recording the ECL signals. For detection, the sensor was immersed in RIF solutions of varying concentrations, and the corresponding ECL responses were recorded. Real sample analysis was conducted by RIF determination in RIF eye drop samples and seawater using the standard addition method. RIF eye drop samples (RIF concentration of 607.5 μM, Wujing Pharmaceutical Co., Ltd., Hangzhou, China) were diluted 100-fold with ethanol. Seawater collected from the shallow continental shelf of the East China Sea (Zhoushan, China) was diluted 100 times with PBS (0.01 M, pH 7.4) before use. Then the obtained eye drop samples or seawater were spiked using known concentrations of RIF standard solution and diluted 100-fold with buffer prior to direct analysis.

## 3. Results and Discussion

### 3.1. Construction of the Sensing Platform and RIF Detection Strategy

**Figure 1:**
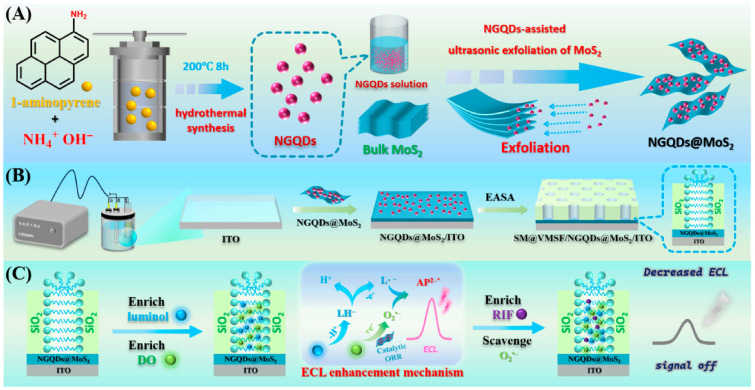
(**A**) Schematic illustration of the synthesis of NGQDs and the preparation process of NGQDs@MoS_2_ nanocomposite. (**B**) Fabrication procedure of the SM@VMSF/NGQDs@MoS_2_/ITO electrode. (**C**) Schematic detection principle based on the enhanced ECL based on enrichment of luminol, DO and RIF, integrated with the catalytic effect of NGQDs@MoS_2_.

Figure 1A illustrates the synthetic route for NGQDs via a one-step hydrothermal process and their subsequent utilization as an intercalation agent to exfoliate MoS_2_ into NGQDs@MoS_2_ composite nanosheets. NGQDs were synthesized via a one-step hydrothermal method using 1-aminopyrene as a precursor in alkaline ammonia medium. The aromatic framework of 1-aminopyrene enables the formation of well-defined sp^2^ carbon domains, while its -NH_2_ groups are in situ integrated as nitrogen dopants (graphitic, pyrrolic, pyridinic), creating abundant surface defects and catalytic sites. Ammonia provides the alkaline environment and serves as an additional nitrogen source. This bottom-up strategy yields uniform NGQDs with rich edge defects and nitrogen-containing groups. The as-prepared NGQDs function as efficient intercalation agents to facilitate the exfoliation of layered MoS_2_. Specifically, the nanoscale NGQDs, characterized by abundant edge defects and nitrogen functionalities, intercalate into the MoS_2_ interlayers. The nitrogen sites and surface hydroxyl groups interact with MoS_2_ via coordination and electrostatic interactions, effectively diminishing the interlayer binding energy. Under ultrasonication, the intercalated NGQDs exert a mechanical wedging effect, promoting interlayer sliding and exfoliation to yield few-layered NGQDs@MoS_2_ composite nanosheets. Throughout this process, NGQDs not only serve as exfoliation aids but also become uniformly anchored on the MoS_2_ surface, forming the nanocomposite.

The fabrication strategy for the sensor (SM@VMSF/NGQDs@MoS_2_/ITO) is depicted in Figure 1B. ITO is used owing to its superior optical transparency, favorable electrical conductivity, electrochemical stability, and cost-effectiveness. Initially, the NGQDs@MoS_2_ layer was modified on the ITO surface via drop-casting (NGQDs@MoS_2_/ITO). Subsequently, a VMSF filled with surfactant micelles was grown in one step on this surface using the EASA method. Specifically, within the CTAB/TEOS sol system, positively charged CTAB micelles adsorb onto the negatively charged ITO substrate via electrostatic interactions. Upon the application of a cathodic current, the in situ reduction of O_2_ or H_2_O at the electrode interface generates OH^−^, creating a localized high-pH gradient. This catalyzes the rapid hydrolysis of TEOS and the formed negatively charged silicate oligomers accumulate at the micelle–substrate interface through electrostatic forces. The resulting film contains surfactant micelles within its nanochannels, yielding the SM@VMSF/NGQDs@MoS_2_/ITO electrode.

Figure 1C illustrates the SM@VMSF/NGQDs@MoS_2_/ITO electrode, which integrates triple enrichment, synergistic catalysis, and specific quenching into a single sensing platform. First, SM@VMSF provides a triple-enrichment effect. Surfactant micelles confined within the nanochannels simultaneously enrich the ECL emitter (luminol), the co-reactant (DO), and the hydrophobic target (RIF). The increase in local concentration of these three species at the electrode interface leads to signal amplification. Second, the NGQDs@MoS_2_ heterostructure exhibits a synergistic catalytic effect. The underlying composite layer enhances electrocatalytic performance by promoting the ORR to efficiently generate ROS, thereby supplying abundant reactive intermediates for the luminol ECL reaction. The combined effects of the enrichment coupled with electrocatalytic ROS generation lead to significantly amplified ECL signals. Finally, RIF quantification is achieved through specific quenching of the ECL signal. This quenching arises from the phenolic hydroxyl groups within RIF molecules, which effectively scavenge the generated ROS, thereby interrupting the ECL reaction pathway. Consequently, the SM@VMSF/NGQDs@MoS_2_/ITO electrode integrates triple enrichment, synergistic catalysis, and specific quenching, providing a robust sensing interface for highly sensitive and selective RIF detection.

### 3.2. Characterization of NGQDs@MoS_2_ Nanocomposite

**Figure 2:**
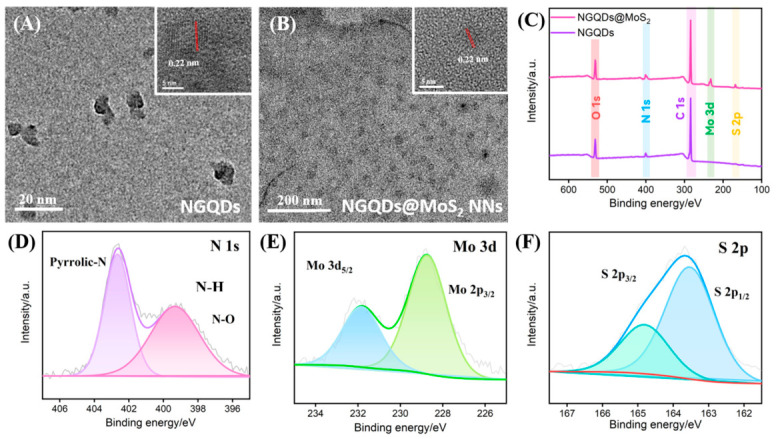
(**A**) TEM images of NGQDs and (**B**) NGQDs@MoS_2_ (insets show the corresponding high-resolution TEM images). (**C**) XPS survey spectra of NGQDs@MoS_2_ and NGQDs. (**D**–**F**) High-resolution XPS (**D**) N 1s, (**E**) Mo 3d, and (**F**) S 2p spectra of NGQDs@MoS_2_.

Figure 2A presents the TEM image of the NGQDs. The NGQDs exhibit a uniform size distribution, with diameters ranging from approximately 3 to 5 nm. The inset displays a HRTEM image, revealing lattice fringes with an interplanar spacing (0.22 nm), which corresponds to the (100) plane of graphene. Figure 2B is a TEM image of the NGQDs@MoS_2_ composite, where dark contrast nanodots are uniformly dispersed across the surface of wrinkled MoS_2_ nanosheets. The HRTEM image in the inset reveals the lattice spacing of NGQDs, confirming the successful immobilization of NGQDs onto the MoS_2_ surface.

XPS was used for the elemental composition and chemical states of NGQDs and NGQDs@MoS_2_, further validating the successful construction of the composite. The survey spectra in Figure 2C indicate that NGQDs are composed of C, N, and O elements. In contrast, the NGQDs@MoS_2_ composite exhibits additional characteristic peaks corresponding to Mo and S, confirming the integration of NGQDs with MoS_2_. The high-resolution N 1s spectrum of NGQDs@MoS_2_ (Figure 2D) displays two distinct peaks (399.7 eV and 402.5 eV), relating to pyrrolic N and N–H bonds from NGQDs, respectively. The Mo 3d spectrum features two peaks of Mo 3d_5/2_ and Mo 3d_3/2_, confirming the presence of the Mo(IV) state (Figure 2E). The S 2p spectrum (Figure 2F) exhibits S 2p_3/2_ and S 2p_1/2_ orbitals with peaks at 163.5 eV and 165.5 eV, respectively, which indicates the formation of Mo-S bonds. These results confirm the formation of the NGQDs@MoS_2_ nanocomposite.

### 3.3. Characterization of SM@VMSF

**Figure 3:**
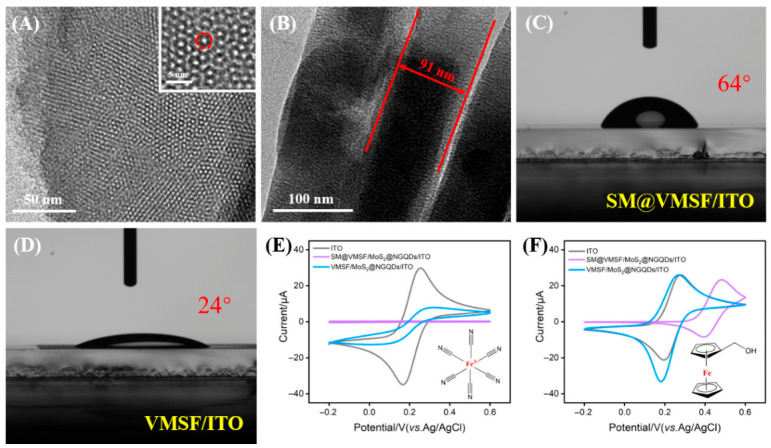
(**A**) Top-view TEM image and (**B**) cross-sectional TEM image of SM@VMSF. (**C**,**D**) Digital photo of contact angle measurements on SM@VMSF/ITO and VMSF/ITO, respectively. (**E**,**F**) Cyclic voltammograms obtained at three electrodes in 0.1 M KCl solution plus (**E**) [Fe(CN)_6_]^3−^ and (**F**) FcMeOH.

The top-view TEM image in Figure 3A reveals that the film prepared by the EASA method exhibits an intact, crack-free structure with highly ordered vertical pore (nanochannel) arrays. The pores display a typical hexagonal arrangement with diameters of approximately 2–3 nm (inset of Figure 3A). The cross-sectional TEM image in Figure 3B demonstrates a uniform film thickness of around 91 nm. Contact angle measurements presented in Figure 3C,D indicate that the contact angle of the electrode with SMs (SM@VMSF/ITO) is 64°, higher than that of the electrode without SMs (VMSF/ITO, 24°). This increase is attributed to the partial exposure of the hydrophobic alkyl chains of micelles within the nanochannels, enhancing the surface hydrophobicity. Such moderate hydrophobicity facilitates the subsequent enrichment of the ECL luminophore (luminol) and the hydrophobic target compound RIF.

The charge selectivity and molecular sieving properties of SM@VMSF were evaluated using different electrochemical probes (Figure 3E,F). For the SM–nanochannel assembly electrode, no significant Faradaic current was observed in the KCl solution containing the [Fe(CN)_6_]^3−^ probe, indicating that micelles confined within the channels effectively block the diffusion of hydrophilic charged ions to the electrode surface. Upon removal of the micelles (VMSF/NGQDs@MoS_2_/ITO), pronounced redox signals were restored for both probes, confirming complete and defect-free coverage of the electrode by the VMSF. Notably, the current response of the VMSF/NGQDs@MoS_2_/ITO toward [Fe(CN)_6_]^3−^ was considerably lower than that of bare ITO (Figure 3E). This result arises from the deprotonation of Si-OH on VMSFs under neutral conditions, rendering the surface negatively charged and electrostatically repelling the anionic probe [47], thereby demonstrating charge-selective permselectivity.

In contrast to charged probes, the neutral hydrophobic probe hydroxymethylferrocene (FcMeOH) exhibited distinct redox peaks at the SM@VMSF/NGQDs@MoS_2_/ITO electrode (Figure 3F), with the oxidation peak potential shifted positively by approximately 50 mV compared to that of bare ITO. This observation suggests that FcMeOH penetrates the hydrophobic core of the micelles via hydrophobic interactions and diffuses to the electrode surface for a redox reaction. The positive shift in oxidation potential directly reflects the thermodynamic partitioning of the probe from the aqueous phase into the micellar phase. These results indicate the enrichment capability of SM@VMSF toward hydrophobic molecules.

### 3.4. ECL Enhancement by SM-Mediated Enrichment of Luminol and DO

**Figure 4:**
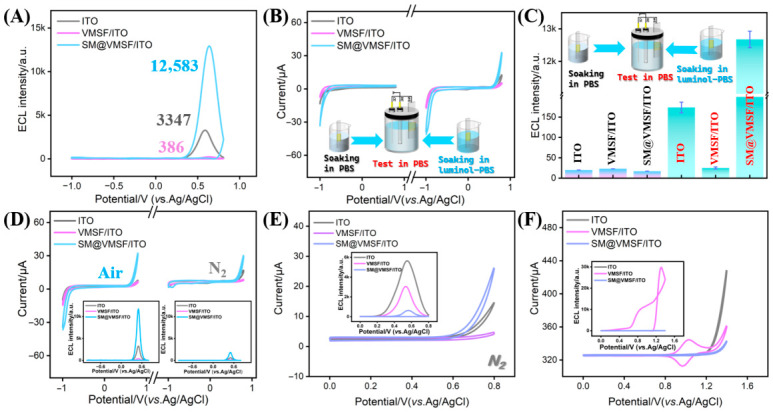
(**A**) ECL curves of three electrodes in luminol solution. (**B**) Cyclic voltammograms and (**C**) ECL signals recorded in PBS after the three types of electrodes were soaked for 15 min in PBS without (**left**) or with (**right**) luminol, followed by rinsing. (**D**) Cyclic voltammograms of different electrodes in PBS with (**left**) and without (**right**) DO. Insets show the corresponding ECL signals. (**E**) Cyclic voltammograms obtained in PBS after the electrodes were soaked in a 100 μM H_2_O_2_ solution and rinsed. The inset displays the corresponding ECL signals. (**F**) Cyclic voltammograms measured in PBS after the electrodes were soaked in a 100 μM Ru(bpy)_3_Cl_2_ solution and rinsed. The inset presents the corresponding ECL signals.

Figure 4A compares the ECL responses of different modified electrodes. The results show that the SM@VMSF/ITO exhibits significantly higher signal intensity compared to VMSF/ITO with open nanochannels. To investigate this SM-induced ECL enhancement, three types of electrodes were immersed for 15 min in PBS either with (experimental group) or without (control group) 100 μM luminol and subsequently transferred to blank PBS for CV and ECL measurements. As shown in Figure 4B, in the control group CV curves (left), only the SM@VMSF/ITO electrode displays a large ORR peak at −1.0 V, with the peak current exceeding those of other electrodes, indicating the capacity of SM to enrich DO. In the experimental group CV curves (right), the SM@VMSF/ITO electrode exhibits a distinct electrochemical oxidation peak of luminol at +0.8 V, confirming SM also enriches luminol. Correspondingly, ECL measurements in Figure 4C reveal that only the SM@VMSF/ITO electrode, after incubation in luminol solution and subsequent transfer to PBS, generates high ECL signals. To clarify the essential role of DO in this ECL system, the electrochemical as well as the ECL signals of the SM@VMSF/ITO electrode were examined under different gas atmospheres. Under N_2_-saturated conditions, the oxygen reduction peak currents at approximately −1.0 V are drastically diminished for all three electrodes (Figure 4D). In contrast, under ambient air, the reduction peak current at SM@VMSF/ITO is remarkably higher than those at VMSF/ITO and bare ITO. Corresponding ECL measurements revealed negligible signal at SM@VMSF/ITO under N_2_-saturated conditions yet this significantly increases upon exposure to air. Thus, DO is essential for the ECL process. These results demonstrate that SM@VMSF enables significant ECL signal amplification through the simultaneous enrichment of both luminol and DO.

### 3.5. Selective Exclusion of Exogenous Co-Reactant and Emitter by SM@VMSF

H_2_O_2_ serves as a typical co-reactant in the luminol ECL system. To investigate whether SM@VMSF can effectively block interference from exogenous H_2_O_2_, ECL signals were recorded under N_2_-saturated conditions in an anodic potential window (0 to +0.8 V), thereby avoiding the interference from the cathodic reduction of residual DO. Under these conditions, any ECL signal originates from H_2_O_2_ acting as the co-reactant for luminol. Figure 4E compares the responses of the three electrodes. CV curves reveal that the SM-containing electrode exhibits the highest anodic oxidation peak current for luminol, attributable to luminol enrichment by the micelles. However, its corresponding ECL signal is the weakest (inset). This result indicates that while luminol can enter the nanochannels and undergo oxidation, the co-reactant H_2_O_2_ is effectively excluded by the SM@VMSF film and cannot reach the electrode surface to participate in the subsequent ECL reaction. This confirms the excellent barrier property of SM@VMSF against the small hydrophilic co-reactant H_2_O_2_.

To further verify the exclusion capability of SM@VMSF toward other types of ECL emitters, the commonly used ECL emitter tris(2,2′-bipyridyl)ruthenium(II) (Ru(bpy)_3_^2+^) was employed with the corresponding co-reactant tripropylamine (TPrA). VMSF/ITO exhibits a pair of well-defined redox peaks (~+1.2 V, Figure 4F), attributed to the free diffusion and electrostatic adsorption of Ru(bpy)_3_^2+^ by the negatively charged VMSF. In contrast, SM@VMSF/ITO displays only a background current from oxygen evolution at approximately +1.4 V, with no characteristic redox peaks of Ru(bpy)_3_^2+^ observed. Corresponding ECL measurements (inset of Figure 4F) demonstrate that the VMSF/ITO electrode generates strong ECL signals, whereas the SM@VMSF/ITO electrode shows a negligible response. These results confirm that SM@VMSF effectively prevents the entry of positively charged Ru(bpy)_3_^2+^ into the nanochannels, further demonstrating its broad selective exclusion capability based on micellar steric hindrance.

### 3.6. ECL Enhancement by NGQDs@MoS_2_ and Interfacial Stability of the Electrode

**Figure 5:**
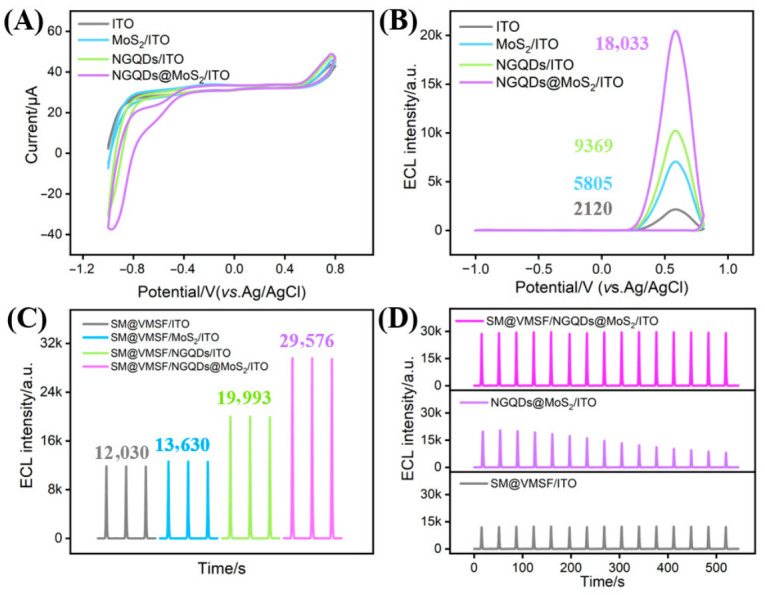
(**A**) Cyclic voltammograms and (**B**) ECL signal–potential profiles recorded at ITO, MoS_2_/ITO, NGQDs/ITO, and NGQDs@MoS_2_/ITO electrodes in PBS containing luminol. (**C**) ECL intensities obtained at the four electrodes upon further modification with SM@VMSF. (**D**) ECL signals of SM@VMSF/ITO, NGQDs@MoS_2_/ITO, and SM@VMSF/NGQDs@MoS_2_/ITO electrodes recorded during consecutive scans.

To investigate the effect of the NGQDs@MoS_2_ composite, the electrochemical and ECL behaviors of modified electrodes with different modification layers were compared. As shown in Figure 5A, within the cathodic potential region of ORR, the NGQDs@MoS_2_/ITO electrode exhibits the highest oxygen reduction current, followed by NGQDs/ITO, while MoS_2_/ITO also displays a higher signal than bare ITO. This confirms that both NGQDs and MoS_2_ are capable of catalyzing the ORR. ECL measurements in Figure 5B reveal that the emission intensity of NGQDs@MoS_2_/ITO exceeds the sum of the ECL intensities obtained from the NGQDs/ITO and MoS_2_/ITO electrodes, indicating a synergistic catalytic effect between the two components. This phenomenon may be ascribed to the fact that NGQDs not only possess good intrinsic ORR catalytic performance but also, when integrated onto the MoS_2_ basal plane, effectively increase the active sites on the MoS_2_ basal plane and promote interfacial charge transfer, thereby generating a synergistic catalytic effect. Within the anodic potential window where luminol electrochemical oxidation occurs, the CV responses of the various electrodes were relatively similar, indicating that the ECL enhancement primarily results from the efficient electrocatalytic reduction of the co-reactant DO. Specifically, this enhancement arises from the promoted conversion of DO to ROS, which accelerates the luminol co-reactant pathway and consequently amplifies the ECL signal. Upon further introduction of these modified electrodes with SM@VMSF, the ECL signals of all the electrodes were boosted (Figure 5C). Notably, the signal at SM@VMSF/NGQDs@MoS_2_/ITO was approximately 2.4-fold higher than that at SM@VMSF/ITO, confirming good compatibility and synergistic enhancement between the nanocomposite and the ordered mesoporous film.

Electrode stability is crucial for sensing applications. Under continuous potential scanning (Figure 5D), SM@VMSF/ITO exhibited stable ECL signals, attributable to the uniform and intact nanostructure of the VMSF film. However, although NGQDs@MoS_2_/ITO initially displayed a high signal, it decayed rapidly during scanning, mainly due to the detachment of the physically adsorbed composite material. In contrast, SM@VMSF/NGQDs@MoS_2_/ITO not only achieved the highest signal intensity but also maintained excellent stability over 15 consecutive scans (RSD = 1.1%). This result indicates that SM@VMSF not only provides an enriching microenvironment but also acts as an effective immobilization matrix, firmly anchoring NGQDs@MoS_2_ onto the electrode surface, thereby achieving both an enhanced ECL signal and a stable interface.

### 3.7. ECL Mechanism of Luminol–DO System

**Figure 6:**
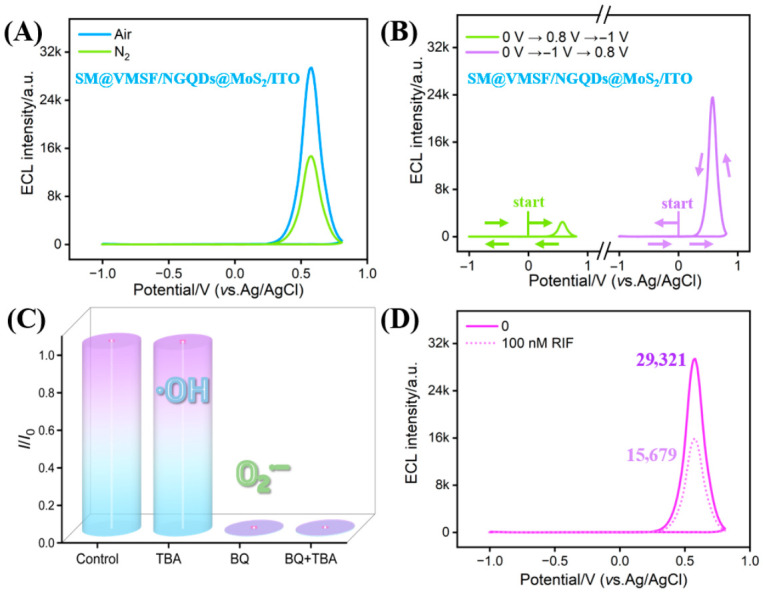
(**A**) ECL responses of SM@VMSF/NGQDs@MoS_2_/ITO under N_2_ or air atmospheres. (**B**) ECL intensity–potential curves obtained with different scan directions. (**C**) ECL response of SM@VMSF/NGQDs@MoS_2_/ITO with various radical scavengers. *I* and *I*_0_ represent ECL signals with or without scavenger, respectively. (**D**) ECL signals of the SM@VMSF/NGQDs@MoS_2_/ITO electrode before and after addition of 100 nM RIF.

Similar to the behavior at the SM@VMSF/ITO electrode, the ECL intensity at the SM@VMSF/NGQDs@MoS_2_/ITO electrode increased with rising O_2_ content in the medium (Figure 6A). DO serves as an essential co-reactant for initiating the ECL signal. The enrichment of DO by SM@VMSF and the catalytic activity of NGQDs@MoS_2_ toward the ORR are critical prerequisites for achieving signal amplification.

The influence of the potential scan direction was investigated. As illustrated in Figure 6B, when employing a cathodic-first scanning sequence (0 V → −1.0 V → +0.8 V), the ECL signal was substantially higher than that obtained with the reverse scan direction (0 V → +0.8 V → −1.0 V). This might result from differences in the lifetimes of key radical intermediates. Specifically, during the cathodic process, DO is reduced to generate ROS, whose relatively long lifetime enable effective accumulation at the electrode interface. In contrast, the luminol anion radical (L^•−^) generated during anodic oxidation possesses a short lifetime. Thus, the cathodic-first scanning order allows the subsequently formed L^•−^ to react sufficiently with the pre-accumulated ROS, maximizing ECL efficiency. Conversely, initiating the scan anodically leads to the decay of L^•−^ prior to ROS generation, resulting in a diminished signal. Based on this observation, all subsequent experiments adopted the cathodic-first scanning mode.

To identify the critical ROS species generated during the cathodic process, benzoquinone (BQ) and tert-butyl alcohol (TBA) were used as scavengers for superoxide anion radicals (O_2_^•−^) [48,49] and hydroxyl radicals (•OH) [50,51], respectively. The results in Figure 6C reveal that the addition of BQ significantly decreased the ECL signal, whereas the introduction of TBA exhibited no significant effect on the ECL signal. The simultaneous addition of both BQ and TBA resulted in a signal reduction comparable to that observed with BQ alone. This indicates that O_2_^•−^ is the key reactive intermediate of the ECL system. Specifically, DO is initially catalytically reduced to O_2_^•−^ by NGQDs@MoS_2_ under cathodic potential. Then, LH^−^ in solution is oxidized at anodic potentials, producing luminol anion radicals (L^•−^). Subsequently, L^•−^ reacts with O_2_^•−^, resulting in the formation of an unstable peroxide intermediate (LO_2_^2−^), which rapidly decomposes into the excited-state dianion (AP^2−^*) with concomitant release of N_2_. The radiative relaxation of AP^2−^* back to the ground state yields ECL emission [52,53,54].

### 3.8. Feasibility of RIF Detection

RIF possesses typical lipophilic characteristics. In the used ECL system (luminol-DO), RIF effectively scavenges the O_2_^•−^ generated, primarily through its phenolic hydroxyl groups. This consumption of key intermediates subsequently reduces the formation of luminol-emitting species, ultimately leading to significant ECL signal quenching. The response of the SM@VMSF/NGQDs@MoS_2_/ITO electrode was recorded upon addition of 100 nM RIF to luminol-containing PBS, as shown in Figure 6D. In the presence of RIF, the ECL signal decreased, indicating the effective quenching of the ECL signal. This result demonstrates the potential for RIF sensing using this electrode platform.

### 3.9. Optimization of Sensor Preparation and RIF Detection Conditions

**Figure 7:**
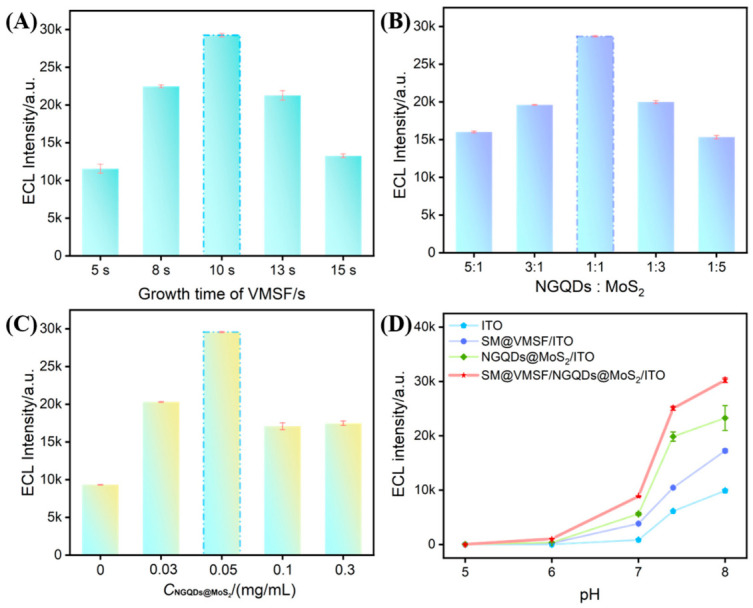
(**A**) ECL signals obtained from SM@VMSF/NGQDs@MoS_2_/ITO electrodes prepared with different SM@VMSF growth times. (**B**) ECL signals of SM@VMSF/NGQDs@MoS_2_/ITO electrodes fabricated with different NGQDs-to-MoS_2_ ratios and (**C**) different concentrations of the NGQDs@MoS_2_ composite. (**D**) ECL signals of different electrodes measured in buffer solutions with various pH values. To prevent the ECL signal from exceeding the measurement range of the instrument at high pH, a relatively low PMT voltage of 550 V was employed.

Key parameters including SM@VMSF growth time, ratio and concentration of NGQDs@MoS_2_, and the pH of the detection solution were optimized. As illustrated in Figure 7A, when the growth time was less than 10 s, the ECL signal exhibited a gradual increase. This is attributed to the insufficient film thickness obtained in the short growth time, resulting in a limited amount of SM loaded within the mesoporous channels and a low ECL intensity. When the growth time exceeded 10 s, the ECL signal decreased with the increase in growth time. This decrease is likely due to the formation of an excessively thick SM@VMSF film, which significantly increases the mass transfer resistance at the sensing interface. Consequently, a growth time of 10 s was selected for subsequent experiments. As shown in Figure 7B, with a fixed concentration of NGQDs, the ECL signal first increased and then decreased as the proportion of MoS_2_ in the composite increased. This trend might be explained by the fact that an initial increase in MoS_2_ content effectively enhances the loading capacity for NGQDs, thereby improving the catalytic performance. However, an excessively high MoS_2_ proportion may lead to a relatively low amount of NGQDs with high catalytic activity, ultimately decreasing ECL performance. The ECL signal intensified when the NGQDs@MoS_2_ concentration increased up to 0.05 mg/mL (Figure 7C). This is because the composite can boost the ECL signal intensity. However, further increasing of the composite concentration resulted in a decreased signal, likely due to the formation of an overly thick modification layer that increases the electron transfer resistance at the interface. Thus, a NGQDs-to-MoS_2_ ratio of 1:1 and a modification concentration of 0.05 mg/mL were chosen. The influence of the buffer pH on the ECL signal was also investigated (Figure 7D). The ECL signal continuously increased as pH increased from 5 to 8. Although the signal continued to rise within the pH range of 7.4 to 8.0, the rate of increase slowed. Considering that a strongly alkaline environment might induce structural changes in the target analyte RIF (e.g., hydrolysis or protonation of amide bonds and phenolic hydroxyl groups), a physiological pH of 7.4 was selected.

### 3.10. Performance of SM@VMSF/NGQDs@MoS_2_/ITO Electrode for RIF Detection

**Figure 8:**
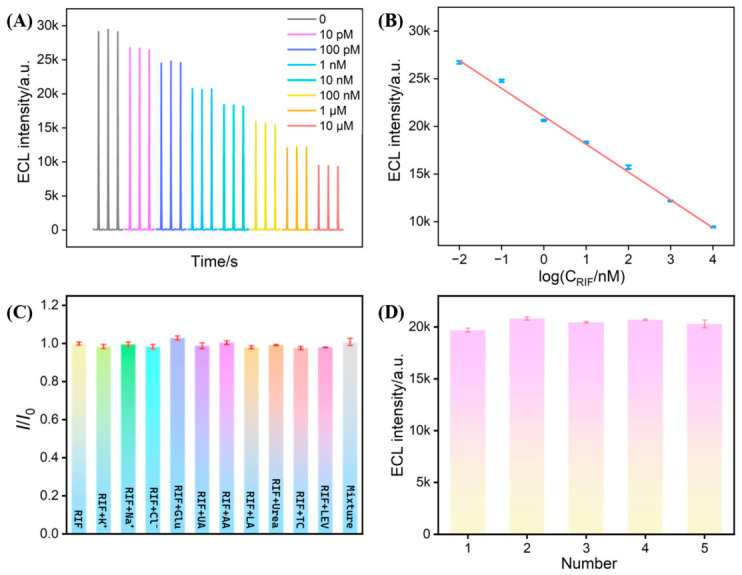
(**A**) ECL responses of the SM@VMSF/NGQDs@MoS_2_/ITO electrode to varying concentrations of RIF. (**B**) Corresponding calibration curve. (**C**) ECL signals of the SM@VMSF/NGQDs@MoS_2_/ITO electrode towards RIF (1 nM) and one of the possible interference substances or their mixtures. The concentrations of K^+^, Cl^−^, Na^+^, Glu, LA, and urea were all 1 μM. The concentrations of AA and UA were 10 μM. The concentrations of TC and LEV were 100 nM. (**D**) ECL signals of five independently fabricated electrodes for the detection of 1 nM RIF.

The quantitative analysis capability of the SM@VMSF/NGQDs@MoS_2_/ITO electrode was evaluated by detecting varying concentrations of RIF via ECL measurements. As depicted in Figure 8A,B, the ECL signal decreases with increasing RIF concentration. A linear relationship was established between ECL intensity (*I*_ECL_) and the logarithm of RIF concentration (log*C*_RIF_) in the range from 10 pM to 10 μM. The corresponding linear regression equation was fitted as *I*_ECL_ = −2927 (±76) log*C*_RIF_ + 21,061 (±201) (*R*^2^ = 0.996). Based on a signal-to-noise ratio of three (S/N = 3), the limit of detection (LOD) for RIF was determined to be 2.5 pM. As shown, our developed SM@VMSF/NGQDs@MoS_2_/ITO sensor exhibited a low LOD value and a wide linear range. This high sensitivity might result from the synergistic effect of the efficient catalysis provided by NGQDs@MoS_2_ and the enrichment capability of the SMs.

To investigate the anti-interference capability of the sensor, its response was challenged by introducing various potential interfering substances commonly present in real samples into the luminol–DO ECL system (Figure 8C). The investigated substances include common ions (K^+^, Cl^−^, Na^+^), high-concentration components (glucose-Glu; urea), redox-active species (uric acid-UA, ascorbic acid-AA, lactic acid-LA) in biological matrices, other antibiotics in clinical practice (tetracycline-TC, levofloxacin-LEV), and their mixtures. Significant quenching of the ECL signal was observed with 1 nM RIF. In contrast, the presence of interfering species at concentrations significantly higher than RIF led to no noticeable effect in the ECL signal. These results demonstrate that SM@VMSF effectively prevents interfering substances from penetrating the mesoporous channels and participating in the reactions at the electrode interface, thereby endowing the sensor with excellent selectivity.

Additionally, the reproducibility of sensor fabrication was investigated. Parallel ECL measurements were conducted in identical test solutions containing 1 nM RIF using five independently fabricated SM@VMSF/NGQDs@MoS_2_/ITO electrodes. As illustrated in Figure 8D, the ECL responses obtained from the five electrodes were highly consistent with a RSD of 1.9%, indicating good reproducibility of the developed sensor.

### 3.11. Analysis of Real Sample

RIF in real samples was performed using standard addition method. The determination of RIF in eye drops or environmental water (seawater) was investigated. The RIF concentration determined by our sensor in eye drops (61.6 nM) is highly similar with that obtained using high-performance liquid chromatography (HPLC, 60.8 nM). The accuracy and repeatability of the detection were assessed by calculating spiked recovery rates and relative standard deviations (RSD, *n* = 5). The recovery rates for RIF ranged from 98.3% to 104% with RSD values between 1.1% and 4.1% (Table 1), demonstrating the precision of the developed sensor for analyzing samples in complex matrices.

## 4. Conclusions

In this study, a nanocatalytic layer composed of NGQDs@MoS_2_ composites was initially fabricated on a low-cost ITO electrode, followed by the in situ growth of a surfactant-modified nanochannel film (SM@VMSF). This approach successfully yielded a sensing interface with nanocatalyst-supported and nanochannel-confined surfactant assemblies. Owing to the synergistic effects between SM@VMSF and NGQDs@MoS_2_, the sensor was applied for the sensitive detection of RIF. The hydrophobic micelles of SM@VMSF enable triple enrichment of the ECL emitter, co-reactant, and target analyte, thereby enhancing ECL signals and detection sensitivity. Additionally, SM@VMSF effectively excludes exogenous co-reactants or other ECL emitters, leading to excellent anti-interference capability to the sensor. The NGQDs@MoS_2_ composite efficiently catalyzes the ORR reaction to generate superoxide anions, which amplifies the ECL signal of luminol–DO. Under optimized conditions, the sensor exhibited a broad detection range and high sensitivity for RIF determination, and it was successfully applied to analyze RIF in real eye drop samples. This work provides a novel approach for the rapid detection of trace RIF in complex matrices and offers new insights into the application of functional mesoporous film and 2D nanocomposites in ECL sensing.

## Figures and Tables

**Table 1:** Detection of RIF in eye drops or seawater using the fabricated ECL sensor.

Sample	Added (nM)	Found (nM)	RSD (%, *n* = 5)	Recovery (%)
RIF eye drops	—	61.6	—	—
	10.0	71.4 (71.5, 72.4, 70.5, 69.2, 73.5)	2.4	98.0
	100	166 (168, 166, 164, 164, 168)	1.1	104
	1000	1046 (1080, 986, 1051, 1093, 1018)	3.8	98.4
Sea water ^a^	10.0	10.1 (10.5, 10.4, 10.1, 9.73, 9.75)	3.2	101
	100	102 (97.5, 101, 102, 107, 101.3)	3.0	102
	1000	1013 (950, 1027, 1049, 1057, 981)	4.1	101

^a^ RIF was not detectable in seawater.

## Data Availability

The data presented in this study are available on request from the corresponding author.
